# Universal Salt Iodisation: Lessons learned from Cambodia for ensuring programme sustainability

**DOI:** 10.1111/mcn.12827

**Published:** 2020-08-24

**Authors:** Karen Codling, Arnaud Laillou, Christiane Rudert, Mam Borath, Jonathan Gorstein

**Affiliations:** ^1^ Regional Coordinator for Southeast Asia and the Pacific, Iodine Global Network Bangkok Thailand; ^2^ Chidl Survival and Development UNICEF Cambodia Phnom Penh Cambodia; ^3^ UNICEF East Asia and Pacific Regional Office Bangkok Thailand; ^4^ National Sub‐Committee for Food Fortification, Ministry of Planning, Government of Cambodia Phnom Penh Cambodia; ^5^ Iodine Global Network Ottawa Ontario Canada

**Keywords:** Cambodia, iodine deficiency, program sustainability, regulatory monitoring and enforcement, salt iodization

## Abstract

Iodine deficiency is the leading cause of preventable intellectual disability in the world, but it has been successfully prevented in most countries through universal salt iodization (USI). In 2011, Cambodia appeared to be an example of this success story, but today, Cambodian women and children are once again iodine deficient. In 2011, Cambodia demonstrated high‐household coverage of adequately iodized salt and had achieved virtual elimination of iodine deficiency in school‐age children. However, this achievement was not sustained because the USI programme was dependent on external funding, and the national government and salt industries had not institutionalized their implementation responsibilities. Recent programmatic efforts, in particular the establishment of a regulatory monitoring and enforcement system, are turning the situation around. Although Cambodia has not yet fully regained the achievements of 2011 (only 55% of tested salt was adequately iodized in 2017 compared with 67% in 2011), the recent steps taken by the government and the salt industry point to greater sustainability of the USI programme and the long‐term prevention of iodine deficiency in children, women, and the general population.

**Key messages**

- Iodine deficiency has the potential to significantly constrain national social and economic development, through the impairment of early child development. However, it has been successfully prevented in most countries through universal salt iodization.
- In Cambodia, external funding contributed to significant achievements in universal salt iodization and elimination of iodine deficiency; however, in the long term, it compromised programme sustainability.
- Experience in Cambodia and elsewhere shows that establishing and ensuring appropriate and effective regulatory monitoring and enforcement of mandatory salt iodization legislation is essential. Without government enforcement, programme sustainability is jeopardized, and the commitment of the private sector is at stake.
- In the context of mandatory, large‐scale fortification initiatives, government monitoring, and enforcement create a safe and supportive environment for fortification and make fortification easily affordable.

## 1 INTRODUCTION

There has been widespread adoption of mandatory universal salt iodization, the iodization of all salt for human consumption, including salt for food processing, throughout the world since the 1990s, and it is one of the most successful public health programmes in the past 30 years (WHO, 2014). A new global database on food fortification programmes indicates that 108 countries currently have mandatory legislation for salt iodization (Global Fortification Data Exchange, 2018). Today, 86% of households worldwide use iodized salt (UNICEF, 2017), and as a result, out of 139 countries with data on population iodine status from the past 15 years, only 19 countries report insufficient iodine intake (Iodine Global Network, 2017). This compares with 110 countries estimated to have insufficient iodine intake in 1993 (WHO, 1999). Although Cambodia is one of the 108 countries with mandatory salt iodization legislation, most recent data from Cambodia indicates that it is one of the small number of countries remaining in the world with insufficient iodine intake.

## 2 UNIVERSAL SALT IODIZATION AND PREVENTION OF IODINE DEFICIENCY IN CAMBODIA

Cambodia, like many other countries around the world, started to implement a salt iodization programme after WHO and UNICEF recommended universal salt iodization as the main strategy to achieve elimination of iodine deficiency disorders in 1993 (UNICEF/WHO Joint Committee on Health Policy, 1994). In 1996, the Royal Government of Cambodia established the National Sub‐Committee for Control of Iodine Deficiency Disorders, with the commitment to achieve iodization of all salt for human and animal consumption, including salt for food processing (Laillou et al., 2016). In 1997, a national survey reported an average national goitre prevalence in primary school children of 12%, indicating mild deficiency, but moderate deficiency and severe deficiency were found in nine and four of the 20 provinces sampled, respectively (Conkle, Carton, Un, & Berdaga, 2013). Salt iodization was initiated in 1999 and UNICEF, U.S. Agency for International Development, and other development partners provided significant support to the programme in the early years, including funding for purchase of equipment, potassium iodate, and extensive public education on iodine deficiency and promotion of iodized salt. Household coverage of iodized salt remained low however, with the 2000 Cambodia Demographic Health Survey measuring it being used in only 13% of households (National Institute of Statistics, Directorate General for Health [Cambodia], and ORC Macro, 2001). In 2003 and 2004, the situation started to change. The Prime Minister passed mandatory salt iodization legislation, and the National Nutrition Council and Ministers of Planning and Commerce issued two “prakas” or implementation regulations. Also, in 2004, 187 independent small‐scale solar salt farmers formed the Salt Producers Community of Kampot and Kep (SPCKK), establishing a system to coordinate the processing, marketing, and sale of salt, including its iodization. As the majority of Cambodia's salt is domestically produced in Kampot and Kep provinces, the SPCKK is currently the sole domestic supplier of raw salt, and the creation of the SPCKK was a key factor in the subsequent success of the salt iodization programme.

**Figure 1:**
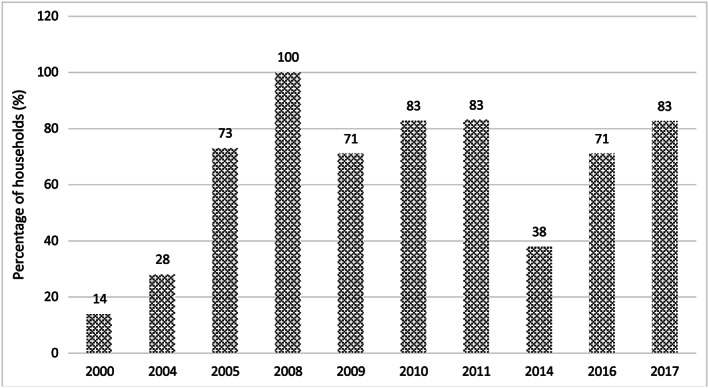
Household use of iodized salt in Cambodia (2000 – 2017). Ref: 2000, 2005, and 2010 Cambodia Demographic Health Survey, 2004 and 2009 Cambodia Socio Economic Survey, 2008 and 2011 Cambodia Survey on Iodine Nutrition, 2014, 2016 and 2017 market survey. All surveys used a rapid test kit to test for the presence of iodine (blue colour appeared) except for the 2014, 2016 and 2017 market surveys which used a quantitative assessment of iodine content; results here reflect salt with'any iodine’ (>0 ppm)

Following the issuance of mandatory salt iodization legislation and the creation of the SPCKK, production of iodized salt increased rapidly, and on average, 82% of households were recorded as having iodized salt between 2005 and 2011 (Figure 1). Although this achievement was noteworthy, the potassium iodate used to fortify the salt throughout this period was paid for with external funding from UNICEF. In 2010, UNICEF ended its support of the potassium iodate, and the SPCKK committed to maintain iodization and purchase sufficient potassium iodate. Little information was generated by the programme after 2011 until UNICEF collaborated with the Ministry of Planning to undertake a market survey to assess iodization compliance in 2014. The survey collected 1,862 samples of salt from major rural and urban markets in all 24 of Cambodia's provinces and recorded that only 38% of samples contained any iodine (Laillou, Mam, Oeurn, & Chea, 2015). Although not representative of national availability of iodized salt, the market survey was the first confirmation that the once successful salt iodization programme had declined. Figure 1 shows the rise and fall in the proportion of households using iodized salt (i.e., salt with any amount of iodine; not necessarily adequately iodized).

The collapse of the programme was further confirmed by an assessment of the iodine status of children 6–59 months by the 2014 Cambodia National Micronutrient Survey, which was implemented in a subset of households sampled in the 2014 Cambodia Demographic Health Survey. The Cambodia National Micronutrient Survey collected spot urine samples from 950 children under 5 and found a median urinary iodine concentration (MUIC) of 72 μg/L, indicative of mild iodine deficiency in this population group (Laillou et al., 2016). This low MUIC in young children in 2014 compares with adequate MUIC levels (236 μg/L in both years) assessed in school‐age children (8–10 years) in 2008 and 2011 when the coverage of iodized salt was still high (National Sub‐Committee for Control of IDD & UNICEF, 2008 and 2011). The same 2014 survey found that women of reproductive age were also deficient with an MUIC of 63 μg/L (Laillou et al., 2016; Table 1).

**Table 1:** MUIC and iodine status of different population groups in Cambodia (2008–2016)

Year	2008	2011	2014	2016
Pop. group	SAC	SAC	WRA	WRA
Sample size	2,329	2,310	736	513
MUIC (μg/L)	236	236	63	74
Iodine status	Adequate	Adequate	Deficient	Deficient
Pop. group			U5	PW
Sample size			950	258
MUIC (μg/L)			72	63
Iodine status			Deficient	Deficient

Ref: 2008 and 2011 Cambodia Iodine Nutrition Survey, 2014 Cambodia National Micronutrient Survey and 2016 Monitoring the Iodine Status of Pregnant and Non‐Pregnant Women in 5 Provinces of Cambodia. Reference for iodine status categories is WHO, 2013. Adequate iodine status: U5, SAC and WRA = 100–199 μg/L; PW = 150–249 μg/L

*Note*. The population of WRA in 2014 were mothers of children under 5 and in 2016 non‐pregnant women of reproductive age

Abbreviations: MUIC, median urinary iodine concentration; PW, pregnant women; SAC, school‐age children; U5, children 6–59 months old; WRA, women of reproductive age; μg/L, micrograms per litre.

**Figure 2:**
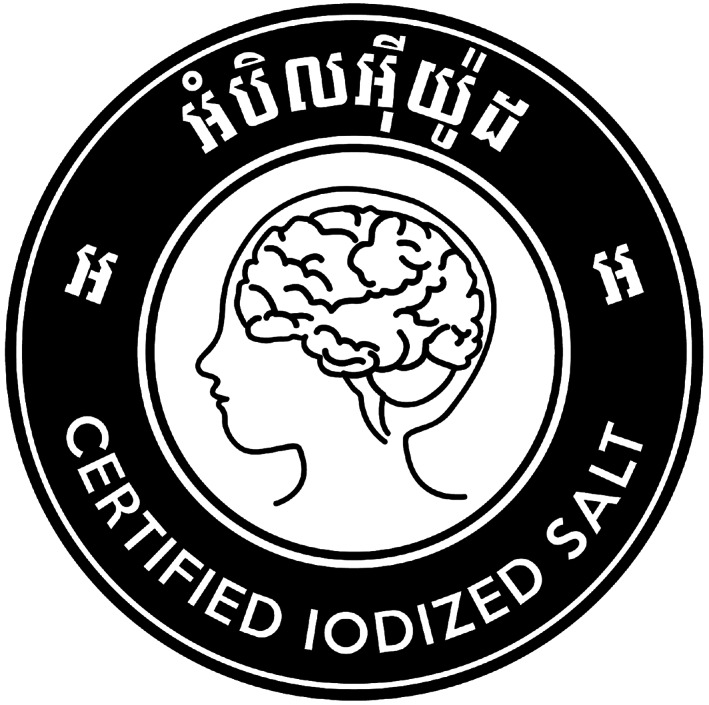
Cambodia's new iodized salt certification logo

Following the alarming salt and urine iodine results of 2014, the government and UNICEF collaborated to get the salt iodization programme back on track. Government and UNICEF action centred on re‐establishing high‐level political commitment and government resources for mandatory salt iodization, identifying and addressing salt industry bottlenecks to iodization and establishing systems for external regulatory monitoring and enforcement funded by government resources. In June 2018, the Government of Cambodia launched *Prakas 85*, a new government policy, which sets salt manufacturing standards and includes the introduction of a new logo (Figure 2) for the certification of iodized salt. The new Prakas requires salt producers to have supplies of potassium iodate, an iodization machine (hand spraying will not be permissible), and an internal quality assurance system, in addition to meeting basic worker health and safety requirements and product quality control. Authorization of factory inspection bodies is ongoing, and a web‐based system for both salt processors and government enforcement agencies to record monitoring results is being established. UNICEF has supported the availability of small‐scale iodization machines and potassium iodate supplies, which can be purchased by the industry through the MoIH on a cost‐recovery basis. These machines and the potassium iodate have been purchased by most of the smaller salt producers that are not part of the SPCKK. The SPCKK has independently purchased 18‐months supply of potassium iodate, although it has yet to address the requirement for a salt iodization machine.

Market surveys in 2016 and 2017 (MoP and UNICEF, 2016 & 2017) suggest the actions are working. As shown in Figures 1 and 2, the proportion of iodized salt has returned to pre‐2014 levels (Figure 1), and the proportion of adequately iodized salt is increasing, although it has yet to reach pre‐2014 levels (Figure 2). Data in Figure 2 are based on quantitative assessments of iodine level whereas those in Figure 1 are based primarily on qualitative (rapid test kit) assessments and present data for “any iodine” only. Meanwhile, an assessment of fish sauce, soya sauce, and fermented fish producers by the UNICEF and the IGN in 2017 was informed that all were using only iodized salt for their production. This was confirmed by tests of samples of salt collected from all producers in Phnom Penh and Battambang where the majority of fish sauce, soya sauce, and fermented fish producers are located (UNICEF and IGN, 2018).

**Figure 3:**
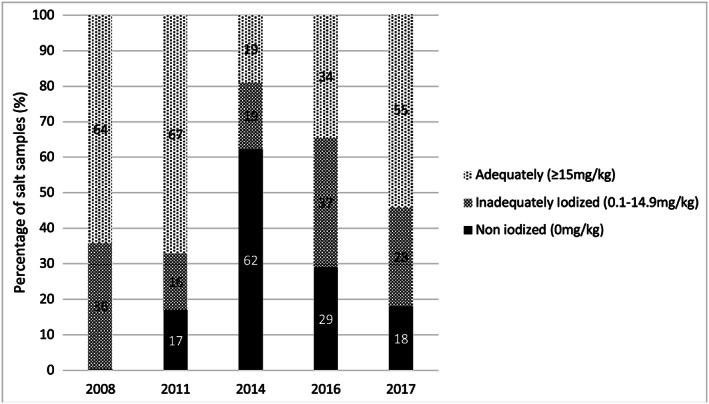
Iodine content of salt in Cambodia (2008 – 2017). Ref: 2008 and 2011 Cambodia Survey on Iodine Nutrition and 2014, 2016, and 2017 market surveys. Results are based on a quantitative assessment of iodine level (sample sizes: 2008:2,329; 2011:2,310; 2014:1,862; 2016:506; 2017:457)

Despite the improvement in coverage of iodized salt, a 2016 subnational survey of five provinces found that both pregnant women (*n* = 258) and reproductive age women (*n* = 513) remain iodine deficient with MUIC levels of 63 and 74 μg/L, respectively (MoP and UNICEF, 2016; Table 1). The reason for the continued poor iodine status, despite an apparent rise in the proportion of salt that is iodized, may lie in the level of iodization. Although the 2016 and 2017 Market Surveys found 71% and 84% of tested salt samples to be iodized, only 34% and 55% contained at least 15 ppm of iodine (Figure 3). Prior to 2014, when the iodization programme was believed to be operating well, quantitative household salt testing indicated that almost 70% of salt samples contained >15 mg/kg of iodine. The 2016 results indicate a significant improvement in the proportion of salt iodized compared with 2014, when only 19% had more than 15 mg/kg, but the small proportion (34%) that contains the minimum amount of iodine for adequate iodine nutrition in 2016 may be the reason for the poor iodine status of pregnant and reproductive age women recorded in the same year (Table 1).

**Figure 4:**
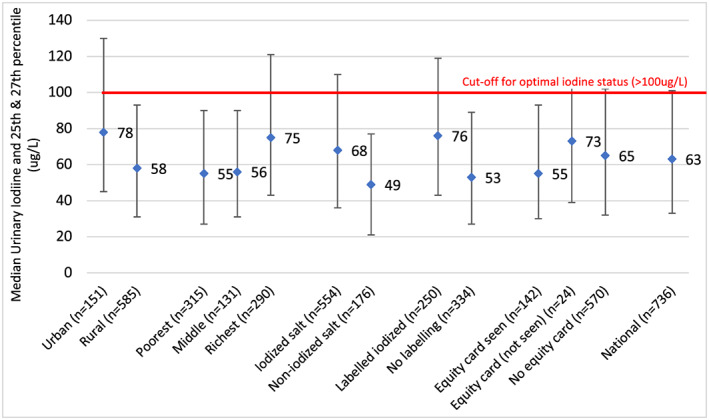
MUIC of mothers of children under 5 (2014). Ref: Laillou A., Sophonneary P., Kuong K., Hong R., Un S., Chamnan C. et al. (2016) Low urinary iodine concentrations among mothers and children in Cambodia. Nutrients, 8. Reference for iodine status categories: WHO, 2013

**Figure 5:**
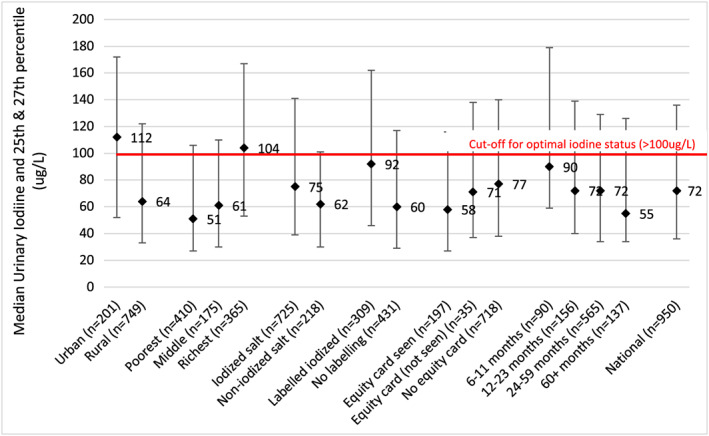
MUIC of children 6‐59 months (2014). Ref: Laillou a., Sophonneary P., Kuong K., Hong R., Un S., Chamnan C. et al. (2016) Low urinary iodine concentrations among mothers and children in Cambodia. Nutrients, 8. Reference for iodine status categories: WHO, 2013

Data on the iodine status, based on MUIC values, of school‐age children, children 6–59 months, women of reproductive age, and pregnant women are shown in Table 1. They reflect iodine deficiency in the population groups assessed in 2014 and 2016, whereas the population group (school‐age children) assessed in 2008 and 2011, when coverage with adequately iodized salt was still above 60%, had adequate iodine status (Figure 3). It is not possible to know, however, what proportion of the Cambodian population is deficient because MUIC cannot be used to identify individuals with iodine deficiency. Analysis of subgroups of the total populations assessed in 2014 can provide a more detailed picture of the situation, although the sample sizes of the subgroups are relatively small, and the interquartile ranges quite wide. Figure 4 illustrates that MUIC was below the optimal level of 100 μg/L in all subpopulation groups of mothers of children under 5 in 2014. Iodine status was significantly lower in rural and poorer women and in those whose salt did not test positive for iodine or was not labelled as iodized. The same pattern is seen in the MUIC values of young children (Figure 5). However, some young child subpopulation groups are not iodine deficient—young children living in urban and richest quintile households, for example. MUIC values by age group of young children indicate that iodine status is highest in the youngest children and declines thereafter, being lowest in the children 60 months and over. This may reflect the fact that after 6 months of age, infants are reliant on dietary iodine intakes rather than iodine stores provided by maternal iodine and breastmilk (Delange, 2004).

**Figure 6:**
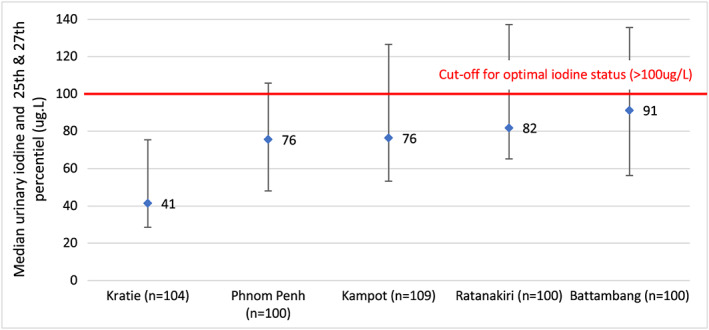
MUIC of non‐pregnant women in 5 provinces (2016). Ref: Ministry of Planning & UNICEF. (2016) monitoring the iodine status of pregnant and non‐pregnant women in 5 provinces of Cambodia, Ministry of Planning, Phnom Penh. Reference for iodine status categories: WHO, 2013

**Figure 7:**
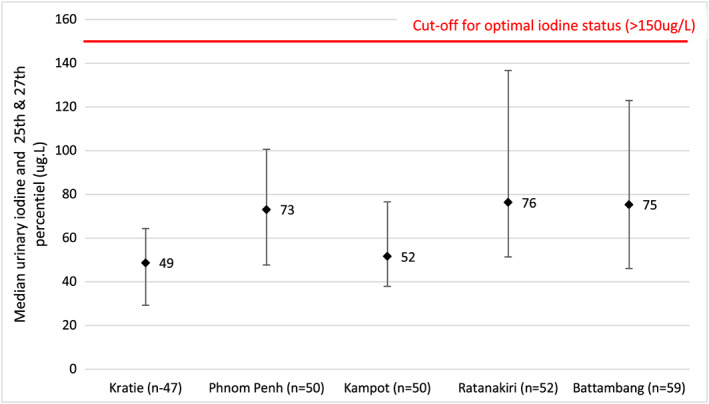
MUIC of pregnant women in 5 provinces (2016). Ref: Ministry of Planning & UNICEF. (2016) monitoring the iodine status of pregnant and non‐pregnant women in 5 provinces of Cambodia, Ministry of Planning, Phnom Penh. Reference for iodine status categories: WHO, 2013

Figures 6 and 7 present the data from the five‐province study of iodine status of pregnant and nonpregnant women in 2016. The provinces in the study were purposively selected to reflect different situations, that is areas of the country receiving imported salt from Lao PDR and Vietnam (Kratie and Ratanakiri), or Thailand (Battambang), for being urban (Phnom Penh) or for being a salt‐producing province (Kampot; MoP and UNICEF, 2016). It is important to recognise that this data are not nationally representative although it has been compared with other nationally representative data in Table 1. The data confirm poor‐iodine status in all the selected provinces. The finding of low iodine status also in Phnom Penh was surprising, as it was expected to have better iodine status in line with the analysis of subpopulation urinary iodine data from 2014 in which urban and richer subpopulations had higher iodine status. (Figures 4 and5).

The dramatic decline in the amount of iodine in salt and subsequent decline in iodine status may be constraining early child development in Cambodia. Iodine is an essential dietary nutrient for the synthesis of thyroid hormones that are critical for normal reproduction, growth, and development. Iodine deficiency therefore leads to impaired synthesis of thyroid hormones, and a spectrum of growth, developmental, and functional abnormalities referred to collectively as iodine deficiency disorders (Delange, 2009). Impaired cognitive development in children occurs even in cases of mild iodine deficiency in the children's mothers (Abel et al., 2017; Bath, Steer, Golding, Emmett, & Payman, 2013; Hynes, Otahal, Hay, & Burgess, 2013), such as is being experienced by Cambodian women today (MoP and UNICEF, 2016), and in particular if women do not enter pregnancy with adequate iodine status, which is also the case for Cambodian women, because of the persistent low availability of adequately iodized salt. Iodine deficiency also has a direct impact on the Cambodian economic growth. A study published in 2016, within the 2014 Cambodian Demographic Health Survey, showed that the second greatest cause of economic losses due to malnutrition was iodine deficiency disorders. If nothing is done to improve the iodine status of its population, the estimated annual economic losses to Cambodia are US$57 million. The different forms of malnutrition studied were estimated to lead to losses of US$266 million or 1.7% of the Gross Domestic Product (Moench‐Pfanner et al., 2016).

## 3 DISCUSSION

Cambodia's experience reflects how rapidly achievements and impacts of salt iodization can deteriorate and the importance of government ownership and programme sustainability. Vietnam's salt iodization suffered a similar deterioration when mandatory salt iodization legislation was made voluntary and government provision of potassium iodate ended (Codling et al., 2015).

A rise in the global price of iodine, and subsequently potassium iodate, as a result of an earthquake and the meltdown of the Daiichi Nuclear Power Plant reactor in Fukushima, Japan in March 2011 has been blamed for the collapse of the salt iodization programme in Cambodia (McNeil, 2017). However, an analysis by the Global Alliance for Improved Nutrition and UNICEF found that although the Fukushima disaster in 2011 exacerbated low availability of global iodine and potassium iodate supplies, iodine prices had been rising since 2003 due to a global shortage of iodine supplies, slowed production due to the economic slowdown in 2008/2009 and production time lags in 2010. Although the price of potassium iodate did rise to US$40–60/kg in 2011 (compared, for example, to US$30 in 2009), it was expected to stabilize by mid‐2012 (GAIN, UNICEF, MI, 2011) and today stands at US$18 (UNICEF Supply Catalogue, 2017).

Whereas the global peak in the price of potassium iodate that occurred in 2011 undoubtedly would have acted as a disincentive to the SPCKK to maintain adequate iodization, a more fundamental reason for the collapse of the programme appears to be inadequate external regulatory monitoring and enforcement of iodization standards by government authorities, such that there was no incentive for iodization or negative fallout from non‐iodization. Unfortunately, at the time of the negotiations for the SPCKK to take over purchase of the potassium iodate, there were not simultaneous efforts to ensure effective government regulatory monitoring. Recent actions of the government, in particular the new requirement for certification of salt producers, has sent the message to salt producers that such enforcement will now start to happen. In response, there has been industry purchase of the potassium iodate and iodization machines made available, suggesting that if regulatory monitoring had been established in 2010, the salt industry may have purchased potassium iodate as per their agreement, avoiding the subsequent decline in iodization and the return of iodine deficiency. Even now however, some producers, including the SPCKK remain complacent, and a major test will come when inspections and enforcement actually start.

Key to sustainable fortification programmes is political commitment to mandatory fortification, including the integration of fortification enforcement into existing systems for food control and streamlined enforcement requirements (e.g., location and frequency of monitoring and short checklist of issues to monitor) to ensure they can be sustainably implemented within existing government resources. Points of production and import are the most efficient points for government to monitor as they are points at which corrective action can be taken; in Cambodia, domestic salt producers, that is, SPCKK and salt boilers, are directly responsible for salt iodization, and imported shipments of salt that do not meet national standards can be turned away at the border. Monitoring and inspection activities should include audits of production and iodization records and inspection of facilities. Quantitative testing of samples of salt should also be included but should not be the primary or only activity, as verification that the production facility has adequate equipment and systems to consistently produce adequately iodized salt is more important and effective than verifying iodization levels in salt collected at the time of a single visit (GF‐TAG, 2018). At the border, inspection of imports can be restricted to verification of paperwork, for example, certificates of conformity, reviewing labels, and random testing (GF‐TAG, 2018). Enforcement of the requirement to use iodized salt in food processing, in particular salty condiments such as fish sauce, which frequently replace household or table salt in the Southeast Asia region, is equally important. Although this aspect of the programme has not been enforced in Cambodia to date, it appears that the programme benefited from the time when all salt was iodized, and non‐iodized salt was not available, creating a situation where food producers had no choice and have since “gotten used” to using iodized salt (UNICEF & IGN, 2018).

As the government has been preparing for enforcement of mandatory salt iodization, for example, through issue of Prakas 85 and preparation for its implementation, some members of the industry are beginning to acquire necessary inputs for quality iodization. The SPCKK has purchased a considerable supply of potassium iodate, enough for 18 months, despite previously claiming inability to pay. Food industries frequently cite the incremental costs of fortification, for fortification equipment and fortificant, as being prohibitive. However, costs of fortification, for example, compared with fluctuations in price of the food vehicle, are usually very small, and in principle, costs of fortification can be passed on to the consumer and spread across the total national food supply such that they are indiscernible to consumers, including low‐income and price‐sensitive consumers. Costs of fortification can only be spread across the total national food supply in the context of mandatory fortification that is enforced however, as enforcement creates an even playing field for all producers. Hence, the best way governments and development agencies can support industries to implement universal fortification is to create a safe and fair environment for fortification through consistent and effective enforcement. Support in the form of free fortificant or donations of equipment may appear to be helpful but simply delays genuine and complete ownership of fortification by the industry and reduces sustainability. Another way government can support fortification is through the removal of import taxes on fortification inputs in order to keep the costs of fortification as low as possible and to facilitate the transfer of fortification costs to consumers if needed. When spread across the entire food supply, costs of fortification of staple foods and condiments appear to be indiscernible by consumers as evidenced by the high coverage of mandatorily fortified foods and condiments, in particular salt.

Finally, improved monitoring of the key indicators of the programme facilitates government oversight and coordination of government implementation. Key indicators of salt iodization programmes include external regulatory monitoring data on compliance and production practices at salt and processed food production facilities and points of import, quantitative assessment of iodine content of household salt, and assessment of iodine status of priority target groups with subnational stratification. Such data should be jointly reviewed by programme stakeholders to identify programme weaknesses and make adjustments to programme design and implementation, such as to fine‐tune standards for iodization, adjust regulatory monitoring requirements or practices, and to identify communities that are not able to access adequately iodized salt, in order to trace back the causes (e.g., inadequately iodized salt from a single importer or producer) and address them.

## 4 CONCLUSION

Salt iodization has proven a highly effective intervention globally to improve iodine nutrition in the general population (Aburto, Abudou, Candeias, & Wu, 2014). It is intended as a long‐term, sustainable strategy and is becoming the industry norm as more and more countries adopt mandatory fortification (Global Fortification Data Exchange, 2018). Iodine deficiency has returned to Cambodia because previous salt iodization implementation was reliant on external funding and is jeopardizing national economic and social development by compromising early child development. Cambodia's experiences are a demonstration that financial contributions of development partners for fortification need to be used strategically to facilitate and institutionalize fortification practices, such as through external monitoring, but not to directly fund them, in order not to jeopardize sustainability and industry and government ownership. Current actions in Cambodia to establish regulatory monitoring systems appear to be working to create a supportive environment for industry to take on their responsibilities in salt iodization with concurrent improvements in availability of iodized salt and hopefully also improvements in iodine status. As key programme actions are no longer reliant on external support, it can be anticipated the programme achievements will be sustained.

## CONFLICTS OF INTEREST

All authors are proponents of mandatory salt iodization as the most effective intervention for the prevention of iodine deficiency, and Arnaud Laillou, Christiane Rudert, and Borath Mam have all been directly involved in supporting and implementing the salt iodization programme in Cambodia.

## CONTRIBUTIONS

This Review Article was conceptualized by Arnaud Laillou and Christiane Rudert. Information on the status of the salt iodization programme in Cambodia was compiled by Arnaud Laillou. Background data collection was undertaken by Karen Codling, and Karen Codling wrote the first draft of the article. Arnaud Laillou, Christiane Rudert, Jonathan Gorstein, and Borath Mam provided critical inputs.
